# Socioeconomic Differences in Global Brain Asymmetry: An Integrative Approach Using Image Similarity Measures and Structural Equation Modeling

**DOI:** 10.21203/rs.3.rs-6933735/v1

**Published:** 2025-07-11

**Authors:** María Eugenia Bernaschini, Grisel Maribel Britos, Adrián Maximiliano Moneta Pizarro, Juan Carlos Bellassai, María Silvia Ojeda

**Affiliations:** 1 Centro de Investigación y Estudios de Matemática, Consejo Nacional de Investigaciones Científicas y Técnicas, Haya de la Torre y Medina Allende S/N, Ciudad Universitaria, X5000HRV, Córdoba, Argentina.; 2 Facultad de Matemática Astronomía Física y Computación, Universidad Nacional de Córdoba, Haya de la Torre y Medina Allende S/N, Ciudad Universitaria, X5000HRV, Córdoba, Argentina.; 3 Facultad de Ciencias Económicas, Universidad Nacional de Córdoba, Bv. Enrique Barros s/n, Ciudad Universitaria, X5000HRV, Córdoba, Argentina.

**Keywords:** brain lateralization, brain MRI, Gradient Magnitude Similarity Deviation, Multiple Indicators Multiple Causes Structural Equation Modeling, socioeconomic status

## Abstract

The typical human brain exhibits a bilateral asymmetry that can be observed in the distribution of grayscale levels in brain magnetic resonance imaging (MRI). This asymmetry plays a crucial role in the development of higher-level cognition and can be influenced by both socioeconomic and biological factors. In this regard, the objective of this article is to investigate the relationship between brain asymmetry and socioeconomic status (SES) as the primary focus, alongside age and sex. To achieve this, we develop a global index of brain asymmetry using a novel methodology that combines two statistical tools in an innovative manner: the Gradient Magnitude Similarity Deviation image similarity measure, which assesses asymmetries in 2D slices of MRI, and Multiple Indicators Multiple Causes Structural Equation Modeling (MIMIC), which integrates the asymmetry of the 2D slices into a single global index. Our study, conducted on a dataset of 132 healthy individuals, reveals a significant association between SES and brain asymmetry, with individuals of higher SES displaying more pronounced asymmetry compared to those of lower SES. Additionally, asymmetry tends to increase with age, and males exhibit higher asymmetry than females. These findings provide new insights into the association between socioeconomic factors and brain asymmetry, highlighting the relevance of SES in relation to structural brain characteristics.

## 1 Introduction

Brain asymmetry is a fundamental feature of human brain structure, shaping cognition, behavior, and other aspects of brain function. While both hemispheres share many similarities, structural and functional differences between them support specialized abilities, such as language, face processing, visuospatial processing, reasoning and handedness (Kong et al., 2018; Rentería, 2012). Understanding this asymmetry provides valuable insights into how the brain optimizes cognitive functioning and adapts to complex tasks.

Some researchers propose that brain asymmetry evolved as a biological adaptation to enhance information processing efficiency (Corballis, 2017). In humans, lateralization is more variable than in apes (Neubauer, Gunz, Scott, Hublin, & Mitteroecker, 2020), leading to greater structural specialization and functional compartmentalization. A refined organization may support a broader range of complex cognitive and behavioral abilities.

While much research has explored the influence of biological factors, such as age and sex, on brain asymmetry, the potential impact of socioeconomic status (SES) remains largely unexplored. SES, which encompasses an individual’s economic and sociological conditions and is typically assessed through education, income, and occupation (Baker, 2014), has been widely associated with various aspects of health and cognition. Individuals with low SES face an elevated risk of mental health challenges, including a higher likelihood of experiencing mental illnesses and an increased vulnerability to suicide (Poeppl et al., 2022). Furthermore, recent research has linked SES to brain structure, with evidence suggesting that individuals with high SES tend to have larger brain volume in several regions of the left hemisphere and smaller volume in some regions of the right hemisphere, whereas the opposite pattern is observed in individuals with low SES (Poeppl et al., 2022). Another study observed elevated gray matter volume within the left dorsol prefrontal cortex in individuals with low SES (Yaple & Yu, 2020). These findings indicate that socioeconomic factors may play a significant role in shaping brain organization, including its asymmetry.

Regarding the measurement of brain asymmetry in MRI, various methodologies have been employed in the literature, including the Pearson correlation coefficient (Ruppert, Teverovskiy, Yu, Falcao, & Liu, 2011), mean square error (Herzog & Magoulas, 2021), volumetric assessments, surface area calculations, and cortical thickness evaluation (Kong et al., 2022).

In this study, we propose a novel approach that combines two statistical tools to quantify global brain asymmetry and examine its relationship with SES: the Gradient Magnitude Similarity Deviation (GMSD) image similarity index and Multiple Indicators Multiple Causes Structural Equation Modeling (MIMIC). GMSD quantifies the similarity between two images, providing a numerical measure of their resemblance based on the gradient magnitude of the image, which captures the local “intensity change strength” at each pixel. MIMIC model, on the other hand, is a powerful statistical technique for modeling complex relationships between observed and latent variables.

This approach enables a comprehensive assessment of brain asymmetry by first evaluating asymmetry on selected 2D slices of MRI scans using the GMSD image similarity measure. The information from individual slices is then integrated into a unified asymmetry index, modeled as a latent variable within the MIMIC framework. By leveraging this methodology, we investigate the relationship between global brain asymmetry and SES as the primary focus, alongside age and sex. This study aims to provide new insights into how socioeconomic factors relate to brain asymmetry, highlighting the importance of considering SES in neuroscience research.

The paper is organized as follows: Section 2 details the materials and methods, including the data repository and participant information. It describes the algorithm used to compute asymmetry in 2D slices and explains the development of the global asymmetry index. Additionally, this section explores the relationship between global asymmetry and both biological factors and socioeconomic status. Section 3 presents the study’s results. Finally, Section 4 provides a general discussion, conclusions, and suggestions for future research directions in brain asymmetry, particularly regarding the impact of socioeconomic status.

## 2 Materials and Methods

### 2.1 Data Repository and Participants

The data used in this article were obtained from the Open Access Series of Imaging Studies (OASIS), a project dedicated to providing the scientific community with open access to brain MRI datasets for studies related to aging, Alzheimer’s disease, and other neurological conditions. The primary goal of the OASIS project is to facilitate future discoveries in both basic and clinical neuroscience by offering datasets that are challenging for individual laboratories to produce independently. The OASIS data were generated by a consortium of academic and research institutions, led by the Knight Alzheimer Disease Research Center at Washington University in St. Louis.

This project provides datasets with preprocessed images, including motion correction and alignment of each image to the Talairach and Tournoux (1988) atlas space using a rigid transformation. The template atlas consists of a combined young-and-old target, previously generated from a representative sample of young subjects and older subjects without dementia. This allows brain images from different individuals to be compared consistently and accurately (spatial standardization). Additionally, the skull was removed, and intensity inhomogeneity caused by magnetic field nonuniformity was corrected (intensity standardization) (Marcus et al., 2007).

In particular, our study employed a cross-sectional dataset comprising 416 subjects, covering adult ages ranging from 18 to 96 years. All subjects in the dataset are right-handed and include both men and women. Notably, among the included subjects aged 60 and above, one hundred have been diagnosed with very mild to mild Alzheimer’s disease (AD).

Additionally, the dataset has supplementary variables relevant to this article, including the age at the time of image acquisition (in years), sex assigned at birth (male or female), and socioeconomic status. The latter was assessed using the Hollingshead Social Position Index, a multidimensional construct that considers not only material wealth but also education and occupation. The Hollingshead Index is categorized into five levels, ranging from 1 (highest) to 5 (lowest). (Hollingshead, 1957).

In alignment with the goals of this study, only data from individuals without a diagnosis of AD and without missing values for supplementary variables were considered. Consequently, the analysis focused on a total of 132 individuals who met these criteria.

Among the 132 individuals included in the study, 72% were women (95) and 28% were men (37). The age range extends from 33 to 94 years, with a mean age of 69. The distribution of socioeconomic status is as follows: 25.8% of participants reported SES 1 (34), 34.8% reported SES 2 (46), 22% reported SES 3 (29) and 17.4% reported SES 4 (23).

### 2.2 Asymmetry Measurement in a 2D Slice

The symmetry axis of a 2D slice is defined as the line that optimally separates the left and right hemispheres of the brain. To build the asymmetry index in each 2D slice, an algorithm is developed that calculates the similarity between the original image and its reflection across various axes. The symmetry axis is determined as the one that maximizes the similarity between both images, and the similarity value corresponding to this axis is considered the brain asymmetry index for that slice. The similarity between the image and its reflection is determined using the image similarity measure known as Gradient Magnitude Similarity Deviation (GMSD). In the following, we provide a brief introduction to GMSD.

#### 2.2.1 Gradient Magnitude Similarity Deviation

Image similarity indices quantify the degree of similarity or dissimilarity between two images and play a crucial role in various image processing and computer vision applications, such as object recognition and quality assessment. Traditional indices like mean square error (MSE) and peak signal-to-noise ratio (PSNR), although widely used, often show poor correlation with human perception (Xue, Zhang, Mou, & Bovik, 2013). To address this limitation, researchers have focused on developing advanced perception-driven image similarity measures. Among these, the Gradient Magnitude Similarity Deviation (GMSD) index stands out as a noteworthy contribution. This measure is also particularly effective in handling noise distortions commonly found in MRI, including Gaussian noise, salt-and-pepper noise, and speckle noise (Ali, 2018; Pappaterra, Ojeda, Landi, & Obed Vallejos, 2021).

GMSD is constructed based on the image gradient magnitude, a numerical measure that quantifies the local “strength” of intensity change within each pixel of the image. This measure can effectively capture intricate local structures. The formal definition is as follows: let A be an image, and (i,j) represent the coordinates of a pixel of A. The magnitude of the gradient (G) is defined as:

(1) $$G_{A}(i,j)=\sqrt{G_{x}{\left(i,j\right)}^{2}+G_{y}{\left(i,j\right)}^{2}},$$

where $G_{x},\;G_{y}$ are the partial derivatives of the intensity function of the image in the horizontal and vertical directions, respectively.

Now, for two images, denoted as A and B, and given the coordinates of a pixel (i,j), the Gradient Magnitude Similarity Map (*GMS*) is defined as:

(2) $$\text{GMS}(i,j)=\frac{2G_{A}(i,j)G_{B}(i,j)+C}{G_{A}{\left(i,j\right)}^{2}+G_{B}{\left(i,j\right)}^{2}+C},$$

where C>0 has been added to obtain numerical stability when the denominator of (2) is too small. This map enables the comparison of gradient magnitudes between the two images. Note that, if A=B then GMS(i,j) equals 1, representing the maximum *GMS* value.

Finally, to define the GMSD, the standard deviation is used as the pooling strategy:

(3) $$\text{GMSD}=\sqrt{\frac{1}{N}\underset{\left(i,j\right)}{\sum }{\left(\text{GMS}(i,j)-\text{GMSM}\right)}^{2}},$$

where N is the total number of pixels in the map and

(4) $$\text{GMSM}=\frac{1}{N}\underset{\left(i,j\right)}{\sum }\text{GMS}(i,j).$$

This standard deviation-based pooling strategy is employed with the consideration that local differences, arising from the diversity of images local structures, significantly influence subjective similarities between images (Xue et al., 2013).

Note that higher values of GMSD imply greater dissimilarity between two images. As such, GMSD serves as a measure for assessing brain asymmetry in individual 2D slices. Further details are provided below, where we describe the algorithm responsible for this task.

#### 2.2.2 Algorithm for calculating asymmetry in a 2D slice

In the following, we detail the steps of the algorithm for calculating the symmetry axis and the asymmetry index in each 2D slice. The algorithm was implemented in Python 3 and executed on Google Colab (the system has a RAM capacity of 12.7 GB and a disk storage space of 107.7 GB). The processing time on average was 8 seconds per image. The source code is available at https://github.com/eugebernas/BrainAsymmetry. The Python libraries used in this work include imageio, matplotlib, numpy, cv2, imutils, PIL (Pillow), itertools, scipy.ndimage.filters, and scipy.fftpack.

**Step 1:** The center of the 2D slice image may not align with the symmetry axis, but the centroid does. Therefore, the initial step involves locating the centroid of the image and adjusting its position so that its center aligns with the centroid. The centroid of an image is the center of mass or the average position of all pixels in the image, weighted by their intensities. It is calculated as the mean coordinates of all pixels in the x and y directions. For a grayscale image, where each pixel has an intensity value, the centroid (Cx,Cy) is calculated using the following formulas:
(5) $$C_{x}=\frac{\sum_{i}\sum_{j}I(i,j)\cdot i}{\sum_{i}\sum_{j}I(i,j)},$$
(6) $$C_{y}=\frac{\sum_{i}\sum_{j}I(i,j)\cdot j}{\sum_{i}\sum_{j}I(i,j)},$$
  where I(i,j) is the intensity of the pixel at coordinates (i,j), and the summations are over all pixel coordinates in the image.
**Step 2:** We preserved the recentered image. However, recognizing that the centroid calculation may not be optimal, we also stored all the images generated by recentering the recentered image by one and two pixels in all directions.
**Step 3:** We rotated all recentered images around their center both to the left and right in steps of 0.5 degrees, up to a maximum of 5 degrees (the step size and the maximum rotation degree were determined empirically^1^). Afterwards, we mirrored each rotated image and saved the pair (rotated image, its reflection).
**Step 4:** For all pairs (rotated image, its reflection), we calculated their similarity using GMSD. We chose the pair that maximizes similarity.
**Step 5:** The symmetry axis is considered as the central vertical axis of the rotated image derived from the pair selected in the preceding step. The asymmetry index of the 2D slice is then defined as the similarity value associated with the previously chosen pair. At this step, the algorithm concludes.

**Fig. 1:**
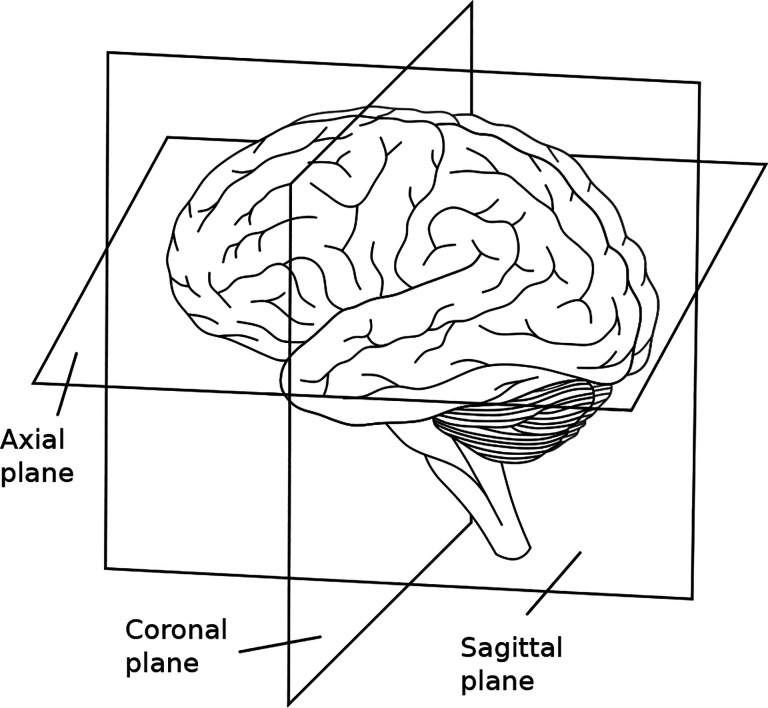
The anatomical planes of the brain include the sagittal, coronal, and axial planes, dividing it into left/right, front/back, and upper/lower portions, respectively

**Fig. 2:**
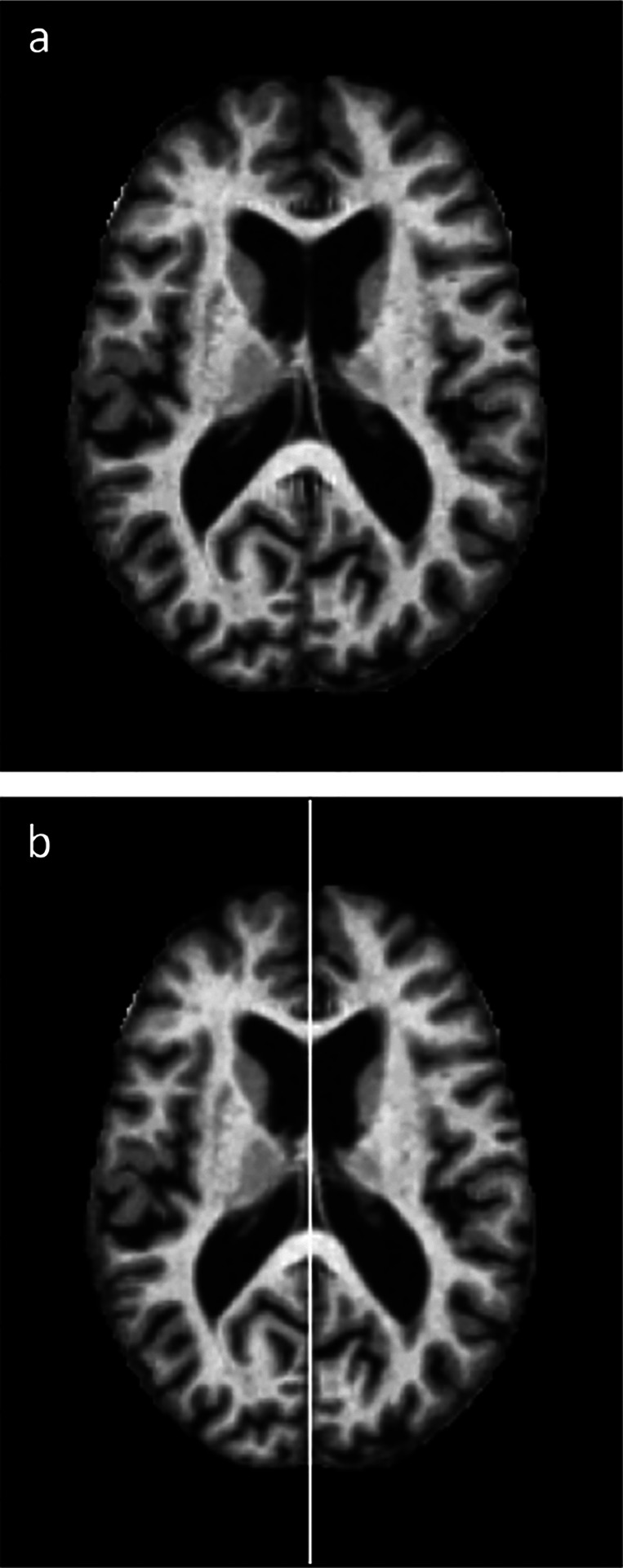
Algorithm result for an axial slice of an image from the database. a. Original image. b. Recentered and rotated image, with the white vertical line indicating the axis of symmetry determined by the algorithm

Each MRI scan in the database is a 3D volume image with dimensions of (176, 208, 176, 1), with the final coordinate denoting the presence of a single color channel. The selected planes for asymmetry calculation were axial slices ((·,·,r,1)), coronal slices ((·,l,·,1)), and one diagonal slice (from the frontal lobe to the cerebellum). In Fig. 1, we illustrate the different anatomical planes of the brain, and in Fig. 2, we present the algorithm’s result for an axial slice of an image from the database.

The symmetry axes in the inferior and superior slices of the brain volume are more ambiguous compared to slices near the center of the brain (Rehman & Lee, 2018). Therefore, only central slices were selected. Additionally, to avoid extreme correlations, we took slices spaced 10 pixels apart. Next, we list the selected axial and coronal 2D slices. Axial 2D slices: (·, ·, 68, 1), (·, ·, 78, 1), (·, ·, 88, 1), (·, ·, 98, 1) and (·, ·, 108, 1). Coronal 2D slices: (·, 84, ·, 1), (·, 94, ·, 1), (·, 104, ·, 1), (·, 114, ·, 1) and (·, 124, ·, 1).

### 2.3 Global Asymmetry Index and MIMIC Structural Equation Modeling

To define a global brain asymmetry index and investigate its association with biological and socioeconomic variables, we estimated a Multiple Indicators Multiple Causes (MIMIC) model within the framework of Structural Equation Modeling (SEM). MIMIC is a multivariate statistical technique that integrates elements of factor analysis and multiple regression to model complex relationships among observed and latent variables.

The MIMIC model consists of two components: the measurement model and the structural model. The measurement model defines the relationship between latent constructs and their observed indicators, assessing how well the indicators reflect the underlying latent variable. The structural model, on the other hand, specifies the associations between the latent variable and observed exogenous variables, capturing the direction and magnitude of these effects.

In our measurement model, Global Asymmetry is conceptualized as a latent variable indicated by several imaging-derived asymmetry measures: diagonal asymmetry, axial asymmetry at slices 68, 78, 88, 98, and 108, and coronal asymmetry at slices 84, 94, 104, 114, and 124. In the structural part of the model, we examined the direct effects of age, sex, and socioeconomic status on Global Asymmetry.

To ensure the validity of the model, we followed the two-step procedure proposed by Anderson and Gerbing (1988). First, we evaluated the measurement model using confirmatory factor analysis (CFA) to confirm that the observed indicators adequately represented the latent construct. Only after establishing an acceptable measurement model did we proceed to assess the structural model, which examined the direct effects of the exogenous variables on Global Asymmetry.

To evaluate model fit, we examined several commonly used fit indices. The Chi-square test assesses the discrepancy between the observed and model-implied covariance matrices; however, it is known to be very stringent, often leading to model rejection even when fit is acceptable (Kline, 2011). The Comparative Fit Index (CFI) and Tucker-Lewis Index (TLI) compare the model to a baseline model, with values above 0.97 indicating excellent fit and values above 0.95 indicating acceptable fit. The Root Mean Square Error of Approximation (RMSEA) is a parsimony-adjusted fit measure that compares models with different numbers of estimated parameters; values below 0.05 are considered good and values below 0.08 are considered acceptable. Its 90% confidence interval and the associated p-value for RMSEA ≤ 0.05 provide additional information. Finally, the Standardized Root Mean Square Residual (SRMR) captures the standardized difference between observed and predicted correlations, with values below 0.05 indicating good fit and values below 0.1 indicating acceptable fit. Recommended cutoff thresholds for these indices can be found in Schermelleh-Engel, Moosbrugger, Müller, et al. (2003).

To improve the model fit, we added residual covariances between specific observed indicators that shared variance not fully explained by the latent factor. This means that, beyond the influence of the latent construct, some pairs of indicators were still correlated—likely due to their spatial or anatomical proximity. Specifically, we allowed covariances among adjacent coronal asymmetry measures (94–104, 104–114 and 114–124) and among adjacent axial asymmetry measures (68–78). These adjustments were theoretically justified—since neighboring brain slices are expected to be more similar—and empirically supported by modification indices (MI). MI values indicate which model parameters, if freely estimated (e.g., allowing residual correlations), would most reduce the discrepancy between the observed and predicted data. Including these residual covariances led to improved model fit, as reflected in better CFI and RMSEA values.

The model was estimated using maximum likelihood (ML) estimation, as implemented in the lavaan package in R (Rosseel, 2012).

## 3 Results

The standardized factor loadings of the measurement model are presented in Table 1. All indicators showed moderate to high loadings (range: 0.64–0.87) and were all statistically significant (p < 0.05), suggesting that they reliably reflect the latent construct of global brain asymmetry. Evaluation of the measurement model yielded highly satisfactory results: Chi-square p-value = 0.05; CFI = 0.99; TLI = 0.98; RMSEA = 0.06; and SRMR = 0.03. These values all indicate an acceptable model fit, supporting the adequacy of the measurement model and justifying the next step: estimating the full structural model.

The standardized regression coefficients from the structural model are shown in Table 2. Socioeconomic status was negatively associated with global asymmetry (β=−0.22,p=0.001), indicating that individuals with fewer socioeconomic resources (i.e., higher SES scores) tend to exhibit less global asymmetry. Age was positively associated with asymmetry (β=0.57,p<0.001), suggesting that brain asymmetry increases with age. Sex also had a significant effect (β=0.40,p<0.001), this implies that, on average, males show higher levels of global brain asymmetry.

Model fit indices are summarized in Table 3. The Chi-square test was significant (p=0.000), which is expected given its strict nature. However, other fit indices indicated satisfactory model fit: the CFI exceeded 0.95, and the TLI was equal to this threshold, the SRMR was below 0.1, and the RMSEA was within an acceptable range. Together, these results support the adequacy of the overall model.

**Fig. 3:**
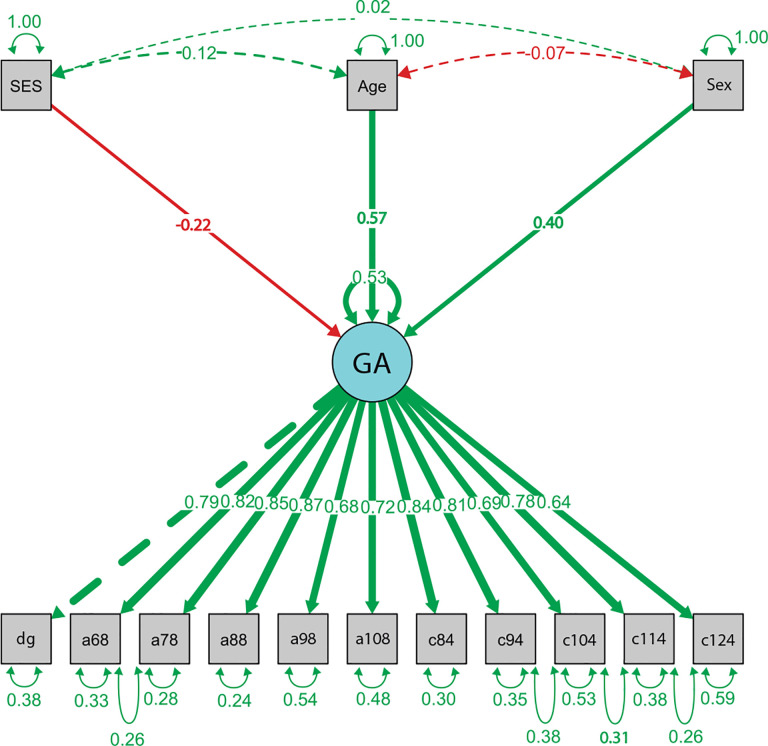
Standardized path diagram. Latent variables are shown as circles; observed variables as rectangles. Arrows indicate the direction of effects. Solid lines represent freely estimated parameters; dashed lines represent fixed or constrained parameters. Numbers on arrows are standardized loadings or path coefficients. Curved arrows pointing back to the same variable represent residual variances (for latent variables) or measurement errors (for observed indicators). Double-headed curved arrows between variables represent covariances

Figure 3 displays the path diagram of the final MIMIC model. The diagram illustrates how the latent construct Global Asymmetry (GA) is measured by a set of observed indicators—axial (a68–a108), coronal (c84–c124), and diagonal (dg) asymmetry measures—and how it is influenced by the exogenous variables SES, age, and sex. Residual covariances among adjacent indicators are included to account for shared variance not explained by the latent factor. This figure provides a graphical summary of the measurement and structural components of the model.

## 4 Discussion

This paper introduces an innovative method for quantifying global brain asymmetry by integrating two statistical tools: the GMSD image similarity measure and Multiple Indicators Multiple Causes Structural Equation Modeling. The choice of GMSD as an image similarity measure was based on its ability to correlate with human perception (Xue et al., 2013). Moreover, it has been observed that GMSD performs well for distortions commonly affecting MRI, such as Gaussian, salt-and-pepper, and speckle noise (Ali, 2018; Pappaterra et al., 2021). On the other hand, the use of MIMIC allowed us to define global asymmetry as a latent variable derived from asymmetries in 2D slices, while simultaneously enabling the evaluation of the association between global asymmetry and biological and socioeconomic variables.

The results indicate that males exhibit greater asymmetry than females, which aligns with previous literature (Guadalupe et al., 2017; Kong et al., 2018; Kovalev, Kruggel, & von Cramon, 2003). Additionally, we found that global asymmetry increases with age, consistent with the findings of Kovalev et al. (2003), Kong et al. (2018) and Guadalupe et al. (2017) in specific brain regions.

### 4.1 Socioeconomic Status and Brain Asymmetry

The key finding of this study is the significant association between socioeconomic status (SES) and global brain asymmetry. While SES has been linked to differences in brain volume and cortical structures (Poeppl et al., 2022; Yaple & Yu, 2020), its impact on global brain asymmetry has remained largely unexplored. Our analysis reveals that individuals with higher SES exhibit greater global asymmetry compared to those with lower SES, adding evidence that SES influences neuroanatomical structure.

Previous studies have shown that SES-related differences in brain structure are often asymmetrical. Poeppl et al. (2022) observed that individuals with high SES tend to have greater brain volume in certain regions of the left hemisphere and reduced volume in various regions of the right hemisphere, whereas the opposite pattern is seen in individuals with low SES. Another study reported increased in activation and gray matter volume in the left dorsol prefrontal cortex in individuals with low SES (Yaple & Yu, 2020).

Beyond structural differences, SES has also been linked to various aspects of cognitive and emotional well-being. Research suggests that individuals with lower SES are more likely to experience mental health challenges (Poeppl et al., 2022) and adolescents report diminished health-related quality of life (Berra, Bernaschini, Mamondi, & Rajmil, 2023).

The observed relationship between SES and brain asymmetry may reflect underlying differences in environmental stimulation, access to resources, and cognitive demands associated with different socioeconomic backgrounds.

### 4.2 Limitations and Future Directions

Understanding how socioeconomic status influences brain architecture is crucial, as it is a modifiable factor that can be addressed through targeted public policies and interventions. Access to education, healthcare, and cognitively enriching experiences may support brain development in ways that promote typical patterns of structural asymmetry. Since altered asymmetry has been linked to various neurodevelopmental conditions (Kong et al., 2018), investigating how SES-related experiences shape the progression of brain asymmetry could shed light on mechanisms of risk and resilience. In addition, given that brain asymmetry is associated with specialized cognitive functions (Corballis, 2017), it is important to explore whether SES-related differences in asymmetry correspond to variations in cognitive performance. More research, especially longitudinal studies, is needed to better understand these associations.

A limitation of this study is that we did not assess brain asymmetry at a regional level, which could yield deeper insights into how SES influences specific neural structures. Future research should focus on measuring asymmetry across distinct brain regions and examining its association with SES. In addition, we plan to explore whether SES-related differences in asymmetry are linked to cognitive performance and neurological conditions, particularly Alzheimer’s disease (AD). Prior evidence suggests a pattern of increased rightward asymmetry in cerebral white matter networks among patients with AD (Yang et al., 2017). Altered brain asymmetry has also been reported in other conditions, including dyslexia, attention-deficit/hyperactivity disorder (ADHD), psychotic disorders, and autism (Kong et al., 2022). Investigating these relationships may help uncover potential connections between SES, brain asymmetry, cognitive function, and neurological health.

Finally, it is worth noting that Kong et al. (2018) did not find significant associations between handedness and brain asymmetries, even when analyzing a large-scale sample comprising several hundred left-handed and several thousand right-handed individuals. Therefore, we do not consider the fact that our dataset consists only of right-handed patients to be a major limitation.

### 4.3 Conclusion

We developed an algorithm to calculate brain asymmetry in 2D MRI slices using GMSD and integrated this information into a single global asymmetry index using MIMIC. This approach allowed us to explore the relationship between brain asymmetry and biological and socioeconomic factors. Our findings reveal a significant association between brain asymmetry and SES, in addition to age and sex, highlighting the impact of socioeconomic conditions on brain structure. These results underscore the importance of considering SES in neuroscience research and suggest that policies aimed at reducing socioeconomic disparities may have implications for brain health and cognitive functioning.

## Figures and Tables

**Table 1:** Standardized factor loadings for the asymmetry latent variable

Indicator	Standardized Loading
diagonal	0.79
axial68	0.82
axial78	0.85
axial88	0.87
axial98	0.68
axial108	0.72
coronal84	0.84
coronal94	0.81
coronal104	0.69
coronal114	0.78
coronal124	0.64

**Table 2:** Standardized regression coefficients

Predictor	Standardized Coefficient	p-value
SES	−0.22	0.001
Age	0.57	< 0.001
Sex	0.40	< 0.001

**Table 3:** Model fit indices

Fit index	Value	Acceptable fit
Chi-square	119.7 (df = 70), p = 0.000	p ≥ 0.05
CFI	0.96	≥ 0.95
TLI	0.95	≥ 0.95
RMSEA	0.07	≤ 0.08
RMSEA 90% CI	[0.050, 0.095]	close to RMSEA
p(RMSEA ≤ 0.05)	0.05	p ≥ 0.05
SRMR	0.06	≤ 0.1

## Data Availability

Data were provided by OASIS: Cross-Sectional: Principal Investigators: D. Marcus, R, Buckner, J, Csernansky J. Morris; P50 AG05681, P01 AG03991, P01 AG026276, R01 AG021910, P20 MH071616, U24 RR021382. The computational routines are available in the repository of GitHub: eugebernas/BrainAsymmetry.

## Footnotes

1 The decrease in step size and the increase in the maximum rotation angle did not produce significantly different results.
